# Beliefs and actual practice of oxygen therapy in the ICU

**DOI:** 10.1186/cc12027

**Published:** 2013-03-19

**Authors:** HJ Helmerhorst, MJ Schultz, PH Van der Voort, E De Jonge, DJ Van Westerloo

**Affiliations:** 1 Leiden University Medical Center, Leiden, the Netherlands; 2 Academic Medical Center, Amsterdam, the Netherlands; 3 Onze Lieve Vrouwe Gasthuis, Amsterdam, the Netherlands

## Introduction

The aim of this study was to compare self-reported beliefs with actual clinical practice of oxygen therapy in the ICU. Hyperoxia is frequently encountered in ventilated patients and prolonged exposure has repeatedly been shown to induce lung injury and (systemic) toxicity.

## Methods

An online questionnaire for ICU clinicians was conducted to investigate beliefs and motives regarding oxygen therapy for critically ill patients. Furthermore, arterial blood gas (ABG) samples and corresponding ventilator settings were retrieved to retrospectively assess objective oxygenation between 1 April 2011 and 31 March 2012 in the ICUs of three teaching hospitals in the Netherlands.

## Results

**Figure 1:**
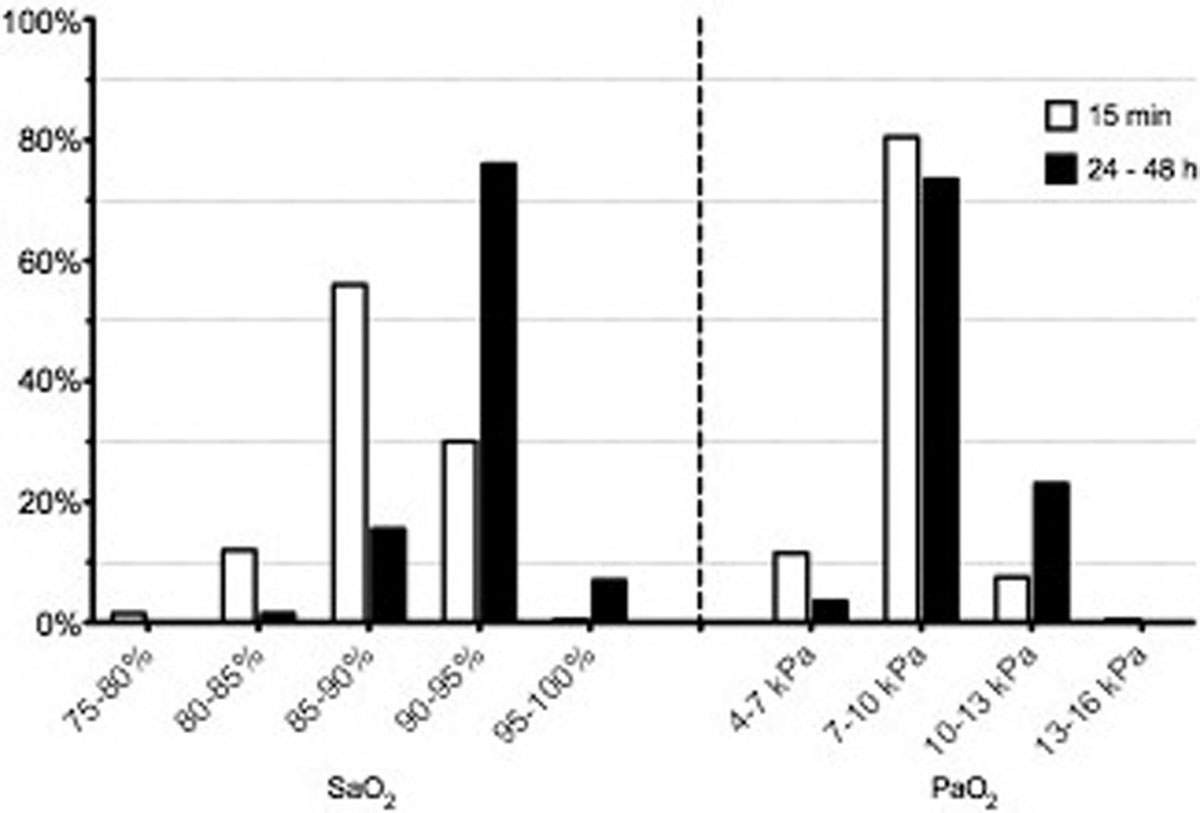


Analyzable questionnaire responses were received from 200 ICU physicians and nurses. The majority of respondents believed that oxygen-induced lung injury is a concern, although barotrauma and volutrauma are generally considered to impose a greater risk in mechanical ventilation. Frequently allowed minimal saturation ranges in the questionnaire were 85 to 95% and 7 to 10 kPa (Figure 1). Self-reported FiO_2 _adjustment in hypothetical patient cases with variable saturation levels was moderately impacted by the underlying clinical condition. To study actual clinical practice, a total of 107,888 ABG samples with corresponding ventilator settings, covering 5,565 patient admissions, were retrieved. Analysis showed a median (IQR) PaO_2 _of 11.7 kPa (9.9 to 14.3), median FiO_2 _was 0.4 (0.4 to 0.5), median PEEP was 5 (5 to 8). A total 63.5% of all PaO_2 _registries were higher than previously suggested oxygenation goals (7.3 to 10.7 kPa) [1]. In 56.8% of cases with PaO_2 _higher than the target range, neither FiO_2 _nor PEEP levels had been lowered when the next ABG sample was taken.

## Conclusion

Most clinicians acknowledge the detrimental effects of prolonged exposure to hyperoxia in the ICU and report a low tolerance for high saturation levels. However, the self-reported intention for conservative oxygen therapy is not consistently expressed in our objective data of actual clinical practice and a large proportion of patients was exposed to high and potentially toxic oxygen levels.
